# Cardiac Magnetic Resonance Imaging in Appraising Myocardial Strain and Biomechanics: A Current Overview

**DOI:** 10.3390/diagnostics13030553

**Published:** 2023-02-02

**Authors:** Alexandru Zlibut, Cosmin Cojocaru, Sebastian Onciul, Lucia Agoston-Coldea

**Affiliations:** 1 Department of Internal Medicine, Iuliu Hatieganu University of Medicine and Pharmacy, 400347 Cluj-Napoca, Romania; 2 Cardiology Department, Emergency Clinical Hospital of Bucharest, 014461 Bucharest, Romania; 3 Faculty of Medicine, “Carol Davila” University of Medicine and Pharmacy, 050474 Bucharest, Romania; 4 Department of Internal Medicine, Cluj County Emergency Hospital, 400347 Cluj-Napoca, Romania

**Keywords:** left ventricle active biomechanics, cardiac magnetic resonance imaging, left ventricle torsion, left ventricle twist and untwist, left ventricle strain

## Abstract

Subclinical alterations in myocardial structure and function occur early during the natural disease course. In contrast, clinically overt signs and symptoms occur during late phases, being associated with worse outcomes. Identification of such subclinical changes is critical for timely diagnosis and accurate management. Hence, implementing cost-effective imaging techniques with accuracy and reproducibility may improve long-term prognosis. A growing body of evidence supports using cardiac magnetic resonance (CMR) to quantify deformation parameters. Tissue-tagging (TT-CMR) and feature-tracking CMR (FT-CMR) can measure longitudinal, circumferential, and radial strains and recent research emphasize their diagnostic and prognostic roles in ischemic heart disease and primary myocardial illnesses. Additionally, these methods can accurately determine LV wringing and functional dynamic geometry parameters, such as LV torsion, twist/untwist, LV sphericity index, and long-axis strain, and several studies have proved their utility in prognostic prediction in various cardiovascular patients. More recently, few yet important studies have suggested the superiority of fast strain-encoded imaging CMR-derived myocardial strain in terms of accuracy and significantly reduced acquisition time, however, more studies need to be carried out to establish its clinical impact. Herein, the current review aims to provide an overview of currently available data regarding the role of CMR in evaluating myocardial strain and biomechanics.

## 1. Background

Myocardial strain and biomechanics are the results of intrinsic normal functioning of the heart, expressing the dynamic interdependency between cardiac structure and its physiology. Usually, in the early stages, heart diseases are clinically silent, often resulting in a delayed diagnosis and poor prognosis. Recent technological advances have developed cardiovascular imaging modalities which are able to thoroughly characterize myocardial tissue and function. Nevertheless, studies evaluating their clinical utility in the diagnosis and prognosis of cardiovascular patients are still sparse. Their close description could provide valuable insights into myocardial functional performance [1]. Briefly, by non-invasively assessing myocardial deformation, one can provide supplementary information regarding disease diagnosis, risk stratification, and prognosis [2].

Cardiac magnetic resonance imaging (CMR) is the gold-standard imaging method used for characterizing heart function and tissue structure, thus providing important information about cardiomyocytes, interstitium, microvasculature, and metabolic abnormalities [3]. Furthermore, MRI has been shown to be useful in post-mortem morphological studies for the study of sudden cardiac death [4]. Increasing evidence is now proving the clinical utility of various CMR methods to determine left ventricle (LV) myocardial strain, torsion, twist, untwist, sphericity index, and long-axis strain determining myocardial strain and biomechanics using various methods [5,6,7,8]. Moreover, several studies have shown good agreement between CMR-based strain and speckle-tracking echocardiography (STE) [6], even though the two methods are still not interchangeable. 

LV myocardial contraction and relaxation are complex phenomena that involve various yet synergistic contractions of all three myocardial layers, thus ensuring hemodynamic stability and optimal LV systolic function. At a glance, there are two ways in which myocardial strain can be outlined: the Lagrangian strain, which provides contractility changes using the own myocardium as a benchmark, and the Eulerian strain, which assesses changes of specific tissue zones with fixed baselines, while the material points differ over time [6]. The disposition of myocardial fibers in the LV is as follows: (1). longitudinal; (2). transversal, with a central distribution and systolic thickening; (3). circumferential, with a circular distribution as viewed from the transversal view of the myocardial [9,10]. The main purpose of these different orientations is to ensure efficient cardiac revolution and maintain global hemodynamics within its normal ranges. In the subendocardial layer, the fibers are longitudinally oriented, from the LV’s base to its apex, while those from the subepicardial layer are inversely directed, from the LV’s apex to its base, and, additionally, the fibers within the middle layer of the myocardium are circumferentially disposed of. All these particularities form a complex multi-layered helical layout, thus guaranteeing adequate longitudinal and circumferential myocardial strain and optimal LV wall shear stress [11].

The main advantage of CMR-determined myocardial strain is in patients with a poor acoustic window in which STE-based ones are not determinable. Additionally, the intra- and inter-observer biases are significantly reduced [6]. Another benefit could be in patients with arrhythmias for whom CMR might provide useful data, especially if single-heartbeat acquisition techniques are used [7]. Nonetheless, there are several disadvantages, such as prolonged evaluation time and extended dorsal decubitus, which in patients with heart failure is often not possible [6]. 

Until now, invasive heart catheterization has been considered the gold-standard method to evaluate cardiac function by using pressure-volume loops, which are valuable markers of myocardial contractility and stroke work, especially by determining cardiac output, end-systolic (ESPVR) and end-diastolic (EDPVR) pressure-volume relationships, or cardiac elastance [12]. Recently, CMR with or without inferior vena cava (IVC) temporary closure has been able to determine ESPVR and EDPVR-derived measurements with comparable accuracy [12,13].

The purpose of this review is to provide an overview of currently available data regarding the clinical role of CMR in evaluating myocardial strain and biomechanics.

## 2. Basics of Myocardial Deformation and Biomechanics

The concept of continuum mechanics in a completely isolated media, along with the properties of cardiac tissue governates the functioning of the cardiovascular system. Thus, myocardial deformation and spatial dynamic geometry are strongly related to these phenomena [14,15]. Moreover, the physical dependency of strain and biomechanics relies on intracellular, extracellular and molecular components of the myocardium. Passive biomechanical properties are ensured by titin, which is an intracellular protein with a high molecular weight that ensures the elastic properties of the myocardial fibers by linking the sarcomeres’ Z lines with the M lines and, thus, preventing the overelongation of these fibers. In this sense, mutations in the titin’s gene have been significantly associated with LV diastolic dysfunction and heart failure [16,17]. On the other hand, active processes, such as deformation, torsion, twist, untwist, and shear stress, are mainly determined by actin and myosin [18]. Other relevant cellular components which are linked to diseased myocardium and heart failure are collagen, which forms complex reticular structures, elastin with its microfibrils of fibulin, and fibrillin, fibronectin, proteoglycans, and glycosaminoglycans [19]. Nonetheless, usually, the myocardium is a soft, heterogenous, anisotropic tissue, which is subject to significant deformations [20], that is based on equation models from the mechanical physics of continuum mechanics. Moreover, several mathematical models, such as the Cauchy stress tensor, the deformation gradient and its Jacobian determiner, and the strain energy density function, have been used to characterize the relation between the LV wall shear stress and myocardial deformation [12,21].

Moreover, myocardial strain and dynamic spatial geometry rely on myocardial contraction forces, which can be assessed using a specific mathematical model that includes the cardiomyocytes’ active tension and calcium ions concentration [22]. LV wall shear stress generates the required forces that strain the cardiomyocytes, and given the situation, they are responsible for the myocardial oxygen mismatch, being easily explained by the law of Laplace [23]. Nevertheless, when it comes to characterizing active biomechanics, there is significant variation depending on the region of interest. When the mid-myocardial layer and the LV’s base are considered, it is recommended to apply different equations to assess the longitudinal and circumferential fibers, while for the LV’s apex, one can use similar mathematical models for both types of myofibers [12].

The primary task of the LV is to ensure continuous blood flow through the vessels during the cardiac cycle. LV function is majorly conditioned by myocardial contraction, end-diastolic filling pressures, and its dynamic geometry, but also by the integrity and correct functioning of heart valves [24,25]. Initially, invasive catheterization was used to accurately describe the heart’s biomechanical physiology, especially by using the curves of ESPVR and EDPVR. Additionally, it has been shown that volume overload increases LV wall shear stress and tension [25]. Furthermore, as postulated in Frank-Starling’s law, increased diastolic filling leads to a higher LV stroke volume due to better functioning of the sarcomeres [26]. Moreover, the maximum elastance, which is the slope between the direct relation between the end-systolic blood volume and the aortic pressure, can accurately assess the contractile ability of the LV, and changes within its inotropy will automatically modify the LV stroke volume [27].

Accordingly, using imaging methods, such as STE and, lately, CMR, deformations of all three myocardial fibers can be globally and regionally assessed, resulting in parameters with important diagnostic and prognostic values: global longitudinal (GLS), circumferential (GCS) and radial (GRS) strains. Various studies have confirmed their paramount roles in diagnosis, risk stratification, and prognosis prediction in many cardiovascular diseases [2]. To identify subclinical LV dysfunction and to subdue the main limitations of standard LV systolic function measurements, international guidelines recommend the comprehensive evaluation of LV strain parameters by echocardiography. These parameters are useful in approaching myocardial ischemia and viability, infraclinical dysfunction in patients with dilated cardiomyopathy, hypertrophic cardiomyopathy, arrhythmogenic cardiomyopathy, cardiac amyloidosis, chemotherapy-induced cardiotoxicity, heart failure, valvular heart diseases, and also in improving the selection of patients who might benefit from cardiac resynchronization therapy [28]. Several studies regarding the importance of LV myocardial strain using STE in various cardiovascular diseases are presented in Table 1 [29,30,31,32,33,34,35,36,37,38,39,40,41,42,43,44,45].

## 3. CMR Methods for Assessing Myocardial Strain and Biomechanics

Although echocardiography is considered the gold-standard imaging technique in assessing LV strain and strain rates, recently, increasing evidence has shown that some CMR techniques are able to appraise myocardial deformation using either specific acquisition variants or post-processing software [6]. Growing evidence has shown their usefulness in patients with ischemic heart disease, various cardiomyopathies, pulmonary hypertension, and congenital heart disease [46]. 

From a technical point of view, the first and foremost magnetic resonance system that has allowed a usable approach to assessing myocardial strain, functioning geometry, and active biomechanics is tissue-tagging CMR (TT-CMR), despite its poor spatial resolution [47]. Subsequently, this shortcoming was overcome by complementary spatial modulation of magnetization, which improved the spatial resolution of the myocardium and the grids [5]. Briefly, in the pre-acquisition phase, tags and lines need to be positioned over the myocardium to track myocardial spatial deformation, angulations, torsion, twist and untwist, and, further, specific sequences are recorded during the LV systole [47]. Nonetheless, to provide an objective and clear upshot, post-acquisition analysis software has been created: FINDTAGS, which quantifies the pixels’ motion during the cardiac cycle, and a more improved one called harmonic phase (HARP), which is fully automated, being the most used for TT-CMR [48]. Still, the main shortcomings of this method comprise prolonged acquisition time and questionable ability to evaluate thin myocardial layers. Likewise, it has been shown that phase-velocity mapping CMR, the method of choice in approaching trans-valvular flows, could become another option for myocardial deformation and biomechanics assessment. By evaluating the spatial differences between each myocardial pixel, phase-velocity mapping CMR can provide all three deformation parameters, through a single breath-hold, by measuring the dynamic differences between pixels [49]. Moreover, fast cine displacement encoding with stimulated echoes (DENSE) uses balanced standard steady-state free precession (b-SSFP) CMR to encode myocardial tissue displacements with intrinsic phase correction to evaluate myocardial deformation. Nonetheless, its main limitation is that it cannot completely assess during the full cardiac cycle [50].

Furthermore, fast Strain-Encoding (fast-SENC) is a valuable CMR technique that uses myocardial magnetization tags, but the main difference from TT-CMR is that the tags are parallelly overlaid on the myocardium, allowing the evaluation of longitudinal and circumferential strain, while radial deformation remains unfortunately unquantifiable [6]. Fast-SENC has increased accuracy and significantly lowered acquisition time due to single-heartbeat free breathing, thus providing increased spatial resolution, as compared to other CMR methods, having also increased ability in detecting a wide range of cardiovascular illnesses [51].

On the other hand, a post-processing software package that could be applied to standard b-SSFP-CMR would hypothetically be the most convenient option to assess LV strain and biomechanics. FT-CMR is an optical flow magnetic resonance method, being derived from the technique which evaluates the motion of fluids. With proper optimization and adjustments, FT-CMR images are comparable to those obtained using speckle-tracking echocardiography and being applicable to standard cine-CMR, it might become highly usable soon [7], but optimization studies need to be further conducted.

## 4. Clinical Utility of CMR in Assessing LV Myocardial Strain

### 4.1. LV Myocardial Strain by CMR in Normal Individuals

Increasing evidence supports the role of CMR in assessing LV myocardial strain in different categories of patients. FT-CMR can determine LV strain measurements in both 2-dimensional and 3-dimensional approaches, with the latter requiring more studies for appropriate validation [7]. It has been reported that the normal values for FT-CMR were −21.3 ± 4.8% for GLS, −26.1 ± 3.8% for GCS, and 39.8 ± 8.3% for GRS [52], while the global rather than regional strain parameters, performed better in terms of reproducibility [53,54]. With a view to validate LV strain analysis by CMR, substantiation research using STE has recently been conducted. In a research paper that compared FT-CMR and strain-encoding (SENC)-CMR with STE, GLS, and GCS determined by both CMR methods had good performances in terms of inter-modality agreement [55]. By comparing FT-CMR with fast-SENC in healthy individuals, it was shown that all three strains had lower values in males than in females, with age being a minor but slightly notable determinant for their variation. In addition, fast-SENC reported a significantly higher value for GLS (−20.3 ± 1.8%) than FT-CMR (−16.9 ± 1.8%), whereas those of GCS were similar (−19.2 ± 2.1% vs. −19.2 ± 1.8%) [56]. Therefore, normal myocardial strain values determined by CMR vary widely depending on various clinical and technical parameters. In the study conducted by Pierpaolo et al., which compared the agreement between manually traced strain and FT-CMR, they have shown poor agreement between the two methods, especially for GLS and GRS [57]. Other variabilities in terms of normal strain values and CMR techniques are presented in Table 2 [58,59,60,61].

Recently, an interesting article sought to assay the capacity and accuracy of fast-SENC to evaluate LV volumes, function, and mass. Almost all the following measurements were precisely determined using fast-SENC, requiring under two minutes of the total study time and being way faster than standard cine-CMR. Nevertheless, LV end-diastolic mass was underestimated by 7% [62]. Furthermore, in another study that aimed to test the accuracy of fast-SENC-based LV myocardial strain, it has been shown that the intra- and inter-observer reproducibility of this CMR method was excellent in terms of LV myocardial functioning assessment [63]. Further studies that could expand the examination in acquiring LV myocardial strain as well might be further conducted, being a very rapid CMR technique. 

### 4.2. LV Myocardial Strain by CMR in Various Cardiovascular Diseases

In a recently published systematic review that evaluated the impact of GLS by both echocardiography and CMR in patients with acute myocardial infarction, it was shown that the latter technique exhibited major advantages in matters of tissue characterization and resolution, regardless of the acoustic window. Nonetheless, larger cohort studies are needed to objectify the real incremental prognostic value that might be deployed by CMR in terms of LV strain characterization [64]. Moreover, a clinical-based study conducted on 232 patients with ST-elevated myocardial infarction searched to appraise the ability of LV myocardial strain determined by FT-CMR in predicting LV post-infarction remodeling. All three global deformation measurements were associated with adverse myocardial remodeling, although only GLS was an independent predictor for it after the adjustment for imaging covariates. Furthermore, a GLS of over −14% increased the risk of adverse remodeling 4 times, with an odds ratio of 4.16, *p* = 0.005, and provided significant incremental predicting value for it [65]. Similarly, the same findings in terms of GLS were also reported by Cha [66]. Likewise, the role of GCS as an independent predictor for late LV myocardial remodeling after myocardial infarction has been proved in the study of Holmes et al. [67]. 

Latterly, fast-SENC is gaining more and more ground even in patients with ischemic heart disease, due to its rapidity and reproducibility. In a recently published study, which sought to compare fast-SENC and FT-CMR with STE in patients with acute myocardial infarction, El-Saadi et al., have shown that in terms of GLS, fast-SENC provided higher values than FT-CMR, but without any statistical significance as compared to STE. Moreover, for GCS, the parameters determined by fast-SENC were almost equal to FT-CMR, while as concerns the regional strain in the infarct-related artery, fast-SENC had a significantly higher area under the curve in properly identifying the injured myocardial segments, in contrast with FT-CMR [68]. Furthermore, Fong et al., conducted a systematic review and meta-analysis in which they compared the utility of GLS in patients with both ischemic and non-ischemic dilated cardiomyopathy. They found GLS as a prognostic predictor for mortality in both groups of patients, however, its predictive ability was lower in those with LVEF of under 30% [69].

In patients with dilated cardiomyopathy, TT-CMR was able to accurately determine impaired LV strain parameters, even within the early stage of the disease [70]. Moreover, LV deformation measurements by FT-CMR were related to the severity of basal dysfunction, whereas GCS alone predicted the recovery of LV ejection fraction [71]. In another cohort of 210 patients with dilated cardiomyopathy, GLS by FT-CMR was also an independent predictor for cardiac death, heart transplant, and ventricular tachyarrhythmias, overcoming GCS, GRS, LVEF, and biomarkers of heart failure [72]. In the study of Korosoglu et al., conducted on 1169 patients with various cardiovascular diseases, the authors sought to evaluate the ability of a fast-SENC-derived strain to diagnose and stratify heart failure. They have shown that the percentage of myocardial segments with impaired strain was able to better identify patients with subclinical heart failure and to improve their risk stratification than standard LV functional parameters [73]. Additionally, in the FT-CMR-based study conducted on 740 patients with myocarditis, GLS was significantly associated with the occurrence of major adverse cardiovascular events, including ventricular tachyarrhythmias, heart failure hospitalization, and all-cause mortality, and proved to be an independent predictor for them [74].

Moreover, in patients suffering from hypertrophic cardiomyopathy, impaired GCS determined by FT-CMR along with LGE were found as independent predictors of ventricular tachyarrhythmias [75]. In addition, a recently published study that sought to evaluate the ability of LV deformation parameters to differentiate between hypertrophic cardiomyopathy and hypertensive heart disease showed that GLS by FT-CMR significantly discriminated between these two illnesses and was also strongly correlated to LGE, T1-mapping, and LV mass [76]. Similarly, LV deformation parameters determined by fast-SENC-CMR were also able to differentiate between athletes’ hearts, hypertrophic cardiomyopathy, and hypertensive heart disease, respectively [77].

Lastly, the role of the LV strain by CMR to identify subclinical myocardial impairment has been recently appraised. In paediatric patients with end-stage renal disease, GLS, GCS, and GRS by TT-CMR, along with LV ejection fraction and mass, were significantly inflicted, whereas GCS and GRS were associated with poor outcomes [78]. Furthermore, more recently, it was shown that LV strain parameters by FT-CMR significantly improved in pediatric patients with end-stage renal disease 1 year after renal transplantation [79]. Similarly, GLS and GCS by FT-CMR were significantly impaired in patients with rheumatoid arthritis, even though standard LV systolic function parameters remained unmodified. In Table 3 [55,56,65,66,67,68,70,71,72,73,75,77,80,81,82,83,84,85,86] are summarized various CMR-based studies on LV myocardial strain.

## 5. Clinical Utility of CMR in Evaluating LV Biomechanics

### 5.1. LV Wringing Parameters

**Figure 1:**
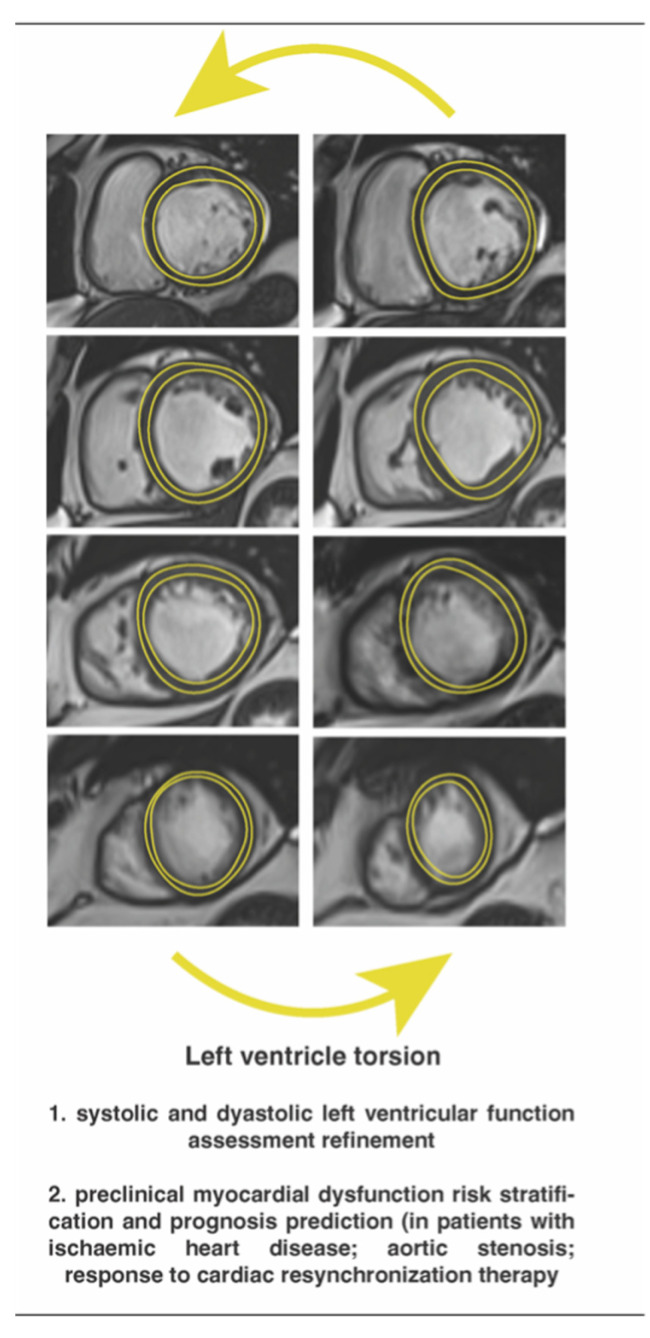
Left ventricle torsion by feature-tracking cardiac magnetic resonance imaging.

Thus far, the association between LV torsion (Figure 1), twist and untwist, and cardiac diseases has been supported by several experimental and clinical CMR studies. At a glance, myocardial fibers strain following base-to-apex and endo-to-epicardium patterns ensure a constant LV circumferential-to-longitudinal shear angle. In this regard, LV torsion is molded during systole when the base and the apex rotate in opposite directions, clockwise and counterclockwise, respectively. This phenomenon results from the normal physiology of the myocardial fibers [87], which is significantly altered in pathological states. In patients with ischemic heart disease, basal rotation was impaired at exertion leading to afflicted LV torsion, while the apical spin remained unchanged, presumably as a compensatory mechanism [88,89]. Conversely, in patients with dilated cardiomyopathy, inverted apical rotation was the main reason for abnormal LV torsion [90].

As previously stated by Rosen et al., when TT-CMR is used to determine LV torsion, the peak systolic LV twist is divided by the inter-slice distance to ensure standardization [91]. Regarding FT-CMR, Kowallick et al., suggested that the best accuracy and feasibility are guaranteed when apical and basal rotations are measured at 25% and 75% of the total LV’s tip-to-base distance [92]. Additionally, if reference values are provided, various post-processing software for CMR can be used to assess LV torsion [93]. Furthermore, this parameter has promising results in risk stratification and prognosis prediction of cardiovascular patients, although studies are just at the beginning. In both apparently healthy elder subjects and diabetics, advanced age and hypertension were associated with higher LV torsion, probably as a result of a balancing mechanism. In addition, it was inversely correlated to the LV sphericity index [94,95]. In contrast, patients with myocardial infarction showed considerably lower LV torsion and the severity of its impairment was significantly associated with an increased risk of cardiovascular death, re-infarction, heart failure hospitalization, and stroke [96]. Withal the standardization of LV torsion to LV long-axis size and radius has led to the development of LV torsion shear angle as a more precise parameter for myocardial remodeling and diastolic function [97]. By normalizing its change rate to analogous variation in LV volume, the LV torsion shear angle was able to accurately identify LV diastolic dysfunction invasively defined as elevated LV end-diastolic pressure and prolonged time of LV relaxation in patients with heart failure and preserved LVEF [98].

LV torsion-to-shortening ratio, which is defined as the ratio between inner wall shortening and torsion at ejection, was developed to accurately characterize endocardial strain and wringing. Essentially, this parameter is a precise marker of subendocardial myocardial impairment, especially in subjects with LV hypertrophy [98]. In patients with aortic valve stenosis (AS), LV torsion-to-shortening ratio determined by TT-CMR was considerably higher when compared to controls, and, in addition, it significantly decreased at three months after aortic valve replacement procedures [99]. FT-CMR has been recently proved to be equally useful in determining impaired LV torsion-to-shortening ratio in patients with AS [100]. Correspondingly, impaired LV torsion-to-shortening ratio and LV torsion have been found even in patients with hypertrophy cardiomyopathy mutation and without clinically overt disease, presumably due to subendocardial malfunction [101].

Over and above, a co-dependency between LV wringing and myocardial scarring has been latterly reported. Intriguingly, reduced LV torsion, along with other abnormal LV systolic parameters, was strongly related to the magnitude of myocardial fibrosis evidenced by Masson’s staining [102]. Due to clinical availability, specific CMR techniques use late gadolinium enhancement (LGE) and native and post-contrast T1-mapping techniques to quantify irreversible replacement and diffuse interstitial fibrosis, respectively [103]. In dilated cardiomyopathy, the presence of LGE was associated with increased basal rotation and decreased apical rotation, which led to defective LV torsion. Additionally, the load of myocardial fibrosis was even higher in those with inverted apical rotation [104]. The presence of LV mid-wall fibrosis, a scarring pattern that is particular for dilated cardiomyopathy, was also closely related to impaired LV torsion and rotation [84]. In contrast, Csecs et al., have failed to prove a significant correlation between the presence and extent of myocardial fibrosis and LV torsion and twist parameters in a well-defined cohort of 239 patients with nonischemic dilated cardiomyopathy, thus suggesting that merely the LV dilation and dysfunction themselves are responsible for impaired LV wringing [105,106]. Therefore, further research is required to correctly ascertain these findings.

As for LV twist, certain evidence concerning the impact of cardiac dysfunction on LV twisting is beginning to emerge to expand the clinical utility of CMR [106]. FT-CMR has been recently shown to have high feasibility and reproducibility in the evaluation of ventricular twist and untwist [54,92]. Therefore, afflicted LV twist was associated with LV enlargement and systolic dysfunction [107]. A recently published systematic review has endorsed the utility of CMR to accurately determine LV untwist [108]. Moreover, Paetsch et al., first demonstrated that in a low-dose dobutamine stress-CMR, LV untwist accurately distinguished patients with ischemic heart disease from controls [109].

### 5.2. LV Functional Dynamic Geometry Measurements

Compelling evidence renders the utility of CMR-derived LV sphericity index in various cardiovascular diseases. Some reports have shown that LV sphericity is inversely associated with LVEF, LV torsion, and mass-to-volume ratio, as well as with both global and regional LV trabeculation indexes [95,110]. Likewise, it was able to correctly identify dilated cardiomyopathy, since it is closely related to increased LV end-systolic volume and decreased LVEF [111]. Correspondingly, by being directly related to sera levels of N-terminal prohormone of brain natriuretic peptide, it may be used for the risk stratification of patients with heart failure [110,112].

**Figure 2:**
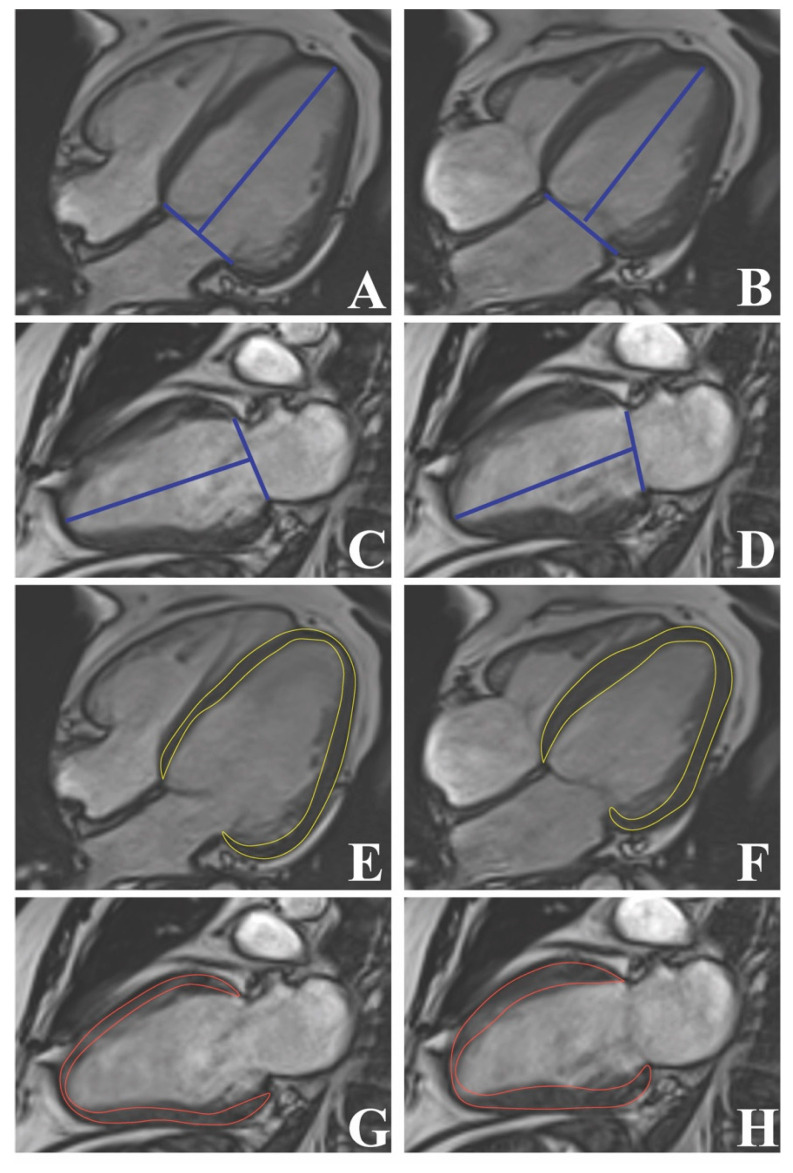
Left ventricular geometry and strain by cine-cardiac magnetic resonance imaging: Left ventricle long-axis strain (**A**–**D**) and sphericity index (**E**–**H**).

Furthermore, the LV sphericity index (Figure 2) is emerging as a novel tool to predict the cardiovascular outcome. In patients with dilated cardiomyopathy, the LV sphericity index significantly predicted major adverse cardiovascular events, including heart failure hospitalization, ventricular tachyarrhythmias, and cardiac death, independent of decreased LVEF and LGE [113,114]. Additionally, in the study of Nakamori et al., the LV sphericity index was an effective marker of appropriate implantable cardioverter defibrillator therapy, thus rightly forecasting ventricular tachyarrhythmias in patients with heart failure and reduced LVEF [115]. Nonetheless, the LV sphericity index might also be useful to predict the occurrence of cardiovascular disease in healthy subjects. In the MESA cohort, the LV sphericity index was found as a strong predictor for the occurrence of ischemic heart disease, heart failure, and atrial fibrillation in initially healthy subjects after 10 years of follow-up [116]. Conclusively, the LV sphericity index is a simple and reproducible parameter, and larger cohort studies should be further conducted to correctly establish its clinical utility.

LV long-axis strain (Figure 2) is a novel indicator of LV systolic function, which can be easily determined by FT-CMR, having high reproducibility and considerable predictive ability [117]. Recently, Leng et al. have shown that standard cine-CMR can also deploy effective and reproducible LV systolic parameters, including LV long-axis strain [118]. Cine-CMR-derived LV long-axis strain has proven non-inferior to FT-CMR-derived one in identifying patients with various cardiomyopathies, being also more time-efficient [119]. Moreover, it was significantly impaired in diabetic patients without clinically overt cardiac disease, even after adjustment for clinical and biological covariates [92]. In the MESA cohort population, impaired LV long-axis strain significantly predicted congestive heart failure, cardiovascular death, stroke, and myocardial infarction, even in subjects without clinically overt cardiovascular illnesses [120]. Likewise, the utility of LV long-axis strain for the prediction of cardiac outcome has also been shown in cardiac amyloidosis, aortic stenosis, and dilated cardiomyopathy [113,117,121]. In addition, it may also improve risk stratification in patients with non-ischemic dilated cardiomyopathy [122]. As for patients with myocardial infarction, impaired LV long-axis strain independently predicted major adverse cardiovascular events at the one-year follow-up [12].

### 5.3. Cardiac Pressure-Volume Loops by CMR

The basic principle of cardiac active biomechanics can be summed up by the relationship between the pressure and volume gradients that develop throughout every cardiac cycle. The close connections between these two physical phenomena have deployed specific pressure-volume curves, which can be used to accurately assess cardiac function. Moreover, specific surrogates of cardiac biodynamics which can precisely estimate myocardial contractility, ventricular-arterial coupling, end-systolic (ESPVR), and end-diastolic (EDPVR) pressure-volume relations can be derived from such measurements. However, the main disadvantage of these measurements is that, until now, they could have been accurately determined only by invasive conductance catheterization [12,123].

**Figure 3:**
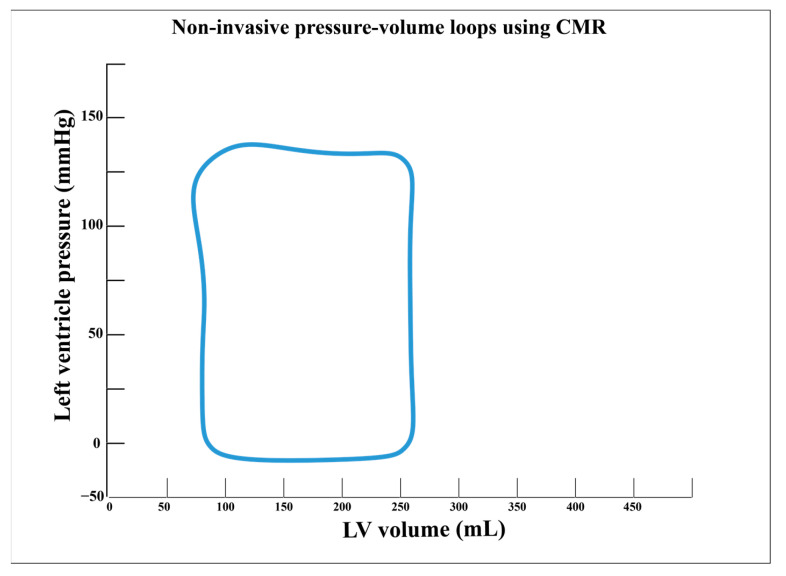
Non-invasive assessment of pressure-volume loops using cardiac magnetic resonance imaging.

Recent studies have begun to deploy hybrid methods that may assess these parameters by combining cine- and velocity-encoded CMR with transient closure of IVC with venous catheters, thus mimicking the preload reduction in cardiac volumes. It was shown that CMR can evaluate the topmost right ventricular pressure during isovolumic normal heartbeats. This may be used to determine ESPVR (Figure 3), yielding it as a potential reliable option to accurately estimate myocardial contractility and ventricular-arterial coupling [124]. Subsequently, Kuehne et al., revealed that venous catheters can be positioned into the pulmonary artery under real-time CMR guidance and, by combining with CMR-determined ventricular volumes and mass, right ventricular pressure-volume loops and ESPVR can be determined. In murine models, they matched these measurements with those determined invasively and found excellent inter-agreements. Moreover, in human subjects, they tested the method on patients with pulmonary hypertension and healthy controls. They found that in the diseased group, cardiac index and ventricular-arterial coupling were significantly afflicted, while ESPVR was increased [125]. 

Likewise, using a similar principle, few pilot studies rendered the utility of CMR to determine EDPVR. In the study of Schmitt et al., pressure-volume curves were initially determined invasively by conductance catheterization. Afterward, using cine- and velocity-encoded CMR along with cardiac pre-load decrease by temporary inferior vena cava occlusion, the authors deployed a hybrid method to estimate EDPVR as a marker of LV stiffness. Not only did they succeed to demonstrate excellent agreement between the two methods, but these measurements were dynamically influenced by pharmacological stress, thus improving diastolic function parameters in a similar manner to those determined strictly by conductance catheterization. Nevertheless, these promising findings require larger studies to validate their clinical utility [126]. Additionally, a murine study proposed a novel method that uses real-time CMR with shorter acquisition timing for cardiac pressure-volume assessment which can be used to continuously determine ventricular volumes, ESPVR, and preload recruitable stroke work as well as eliminate several existing shortcomings. Nonetheless, these methods require larger cohort validation [127]. By the same token, Giao et al., demonstrated the use of real-time CMR in the estimation of ESPVR during inferior vena cava obstruction. They showed that this method provides relevant data regarding LV geometry and regional function and, thus, emphasized the importance of LV shape and segmental biomechanics in maintaining cardiac performance [128].

Moreover, the clinical efficacy of these was first evaluated in murine models by Faragli et al., who sought to assess the relationship between determined LV strain parameters and hemodynamical parameters such as cardiac index, cardiac power output, and ESPVR determined by FT-CMR in various stress conditions. Despite several technical and analytical drawbacks that relatively lowered the statistical power, LV global longitudinal and circular strain were closely related to all LV hemodynamic measurements, regardless of the inotropic state, while LV global longitudinal performed best in assessing LV contractility, similar to LVEF. Therefore, FT-CMR might become a promising technique for evaluating LV hemodynamics; however, future studies are required for the optimization of this method [129]. 

Last but not least, by creating a time-variance elastance mode, Seeman et al., were the first to develop a completely non-invasive method that uses solely CMR and brachial pressure to assess LV pressure-volume loops, thus overcoming the need for IVC occlusion. Firstly, they tested and validated this method in murine models and further confirmed it in human subjects by comparing patients with heart failure with healthy controls [13].

## 6. Future Perspectives

Even though the role of CMR in determining myocardial strain and biomechanical parameters is gaining serious ground, there are still many uncertainties that need to be unraveled. FT-CMR has been proven to be a useful CMR method in assessing myocardial strain, but there is still insufficient evidence in terms of various cardiovascular diseases. Further studies should be conducted in patients with valvular heart disease, such as aortic stenosis or mitral regurgitation, in order to test the predictive ability of myocardial strain parameters in prognosis prediction. Moreover, there are not any available data regarding the role of FT-CMR-derived myocardial strain in patients with cardiac amyloidosis or other infiltrative heart diseases, which might provide information of tremendous importance in risk stratification and prognosis prediction. Further studies could also aim to test the ability of FT-CMR in creating multi-parametric predictive models based on LV wringing parameters in various cardiomyopathies or myocarditis. Additionally, there is little evidence of the role of FT-CMR-derived strain and biomechanics in patients with acute myocarditis.

As for fast-SENC, it is a promising valuable CMR imaging technique that might enter day-to-day practice in the future, but more studies still need to be conducted. Although few studies have shown the superior ability of fast-SENC-derived myocardial strain parameters in risk stratification and prognosis prediction of patients with acute myocardial infarction, there are no studies conducted in patients with various primary myocardial diseases, thus, this could represent a valuable research direction. Moreover, the ability of fast-SENC to deploy LV wringing and functional dynamic geometry parameters represents another uncharted territory.

As for routine clinical applicability, things are still in their infancy. FT-CMR might become a promising option in daily medical practice because it uses standard cine-CMR acquisitions. Additionally, semi-automated or automated software might aid the evaluation. Furthermore, fast-SENC is another promising technique that might significantly reduce the acquisition time for CMR examinations. Nonetheless, due to great disparities in results, which depend on the method of acquisition and data processing, further studies still need to be conducted.

## 7. Conclusions

The role of CMR in assessing LV myocardial strain and biomechanics is beginning to take shape. Recent technological advancements in the field of CMR, such as fast-SENC and FT-CMR, are able to ensure increased accuracy in evaluating myocardial strain, LV wringing, and active geometry parameters and, along with these developments, increasing evidence endorses their future clinical ability. Even though things are just at the beginning, few yet important studies have shown the tremendous potential which lies behind LV strain and biomechanics.

## Figures and Tables

**Table 1:** Speckle-tracking echocardiography studies in evaluating LV GLS.

Authors	Year	Ref	*n*	Illness	Endpoint	GLS	LVEF
Janwanishstaporn et al.	2022	[32]	289	HFimprEF	CVD, HFH	−12.7%	53%
Thellier et al.	2020	[33]	332	AS	ACM	−15%	55%
Goedemans et al.	2018	[34]	143	AMI	ACM, HFH	−14.4%	N/A
Iacoviello et al.	2013	[35]	308	HF	ACM, HFH, CVD, VT	−10.2%	33%
Ersboll et al.	2013	[36]	849	AMI	ACM, CVD, HFH	−14.6%	53.5%
Yingchoncharoen et al.	2012	[37]	79	AS	CVD	−15.2%	63.4%
Munk et al.	2012	[38]	576	AMI	ACM, CVD, HFH, AMI	−14.3%	49.2%
Kearney et al.	2012	[39]	146	AS	ACM, AMI, CVD, HFH, VT	−15%	59%
Dahl et al.	2012	[40]	125	HT	ACM, CVD, HFH	−15.5%	34.1%
Buss et al.	2012	[41]	206	AL	ACM, CVD	−13.1%	51.7%
Bertini et al.	2012	[42]	1060	IHD	CVD, HFH	−11.5%	34%
Woo et al.	2011	[43]	98	AMI	CVD, HFH	−15.8%	56%
Nahum et al.	2010	[44]	125	HF	ACM, CVD, HFH	−8%	31%
Antoni et al.	2010	[45]	659	AMI	ACM, AMI, HFH	−15.3%	46%
Stanton et al.	2009	[46]	546	Various	ACM	−16.6%	58%
Cho et al.	2009	[47]	201	HF	CVD, HFH	−10.5%	34.1%
Lancellotti et al.	2008	[48]	163	AS	CVD, HF	−15.7%	66%

Abbreviations: ACM, all-cause mortality; AMI, acute myocardial infarction; AS, aortic stenosis; CVD, cardiovascular death; GLS, global longitudinal strain; HF, heart failure; HFH, heart failure hospitalization; HFimpEF, heart failure with improved ejection fraction; IHD, ischemic heart disease; LVEF, left ventricle ejection fraction; LVEF, left ventricle; N, number of patients; VT, ventricular tachyarrhythmias.

**Table 2:** LV myocardial strain variations in normal individuals (miscellaneous).

Authors	Year	*n*	Method	Findings
Mangion et al.	2019	88 healthy individuals	FT-CMR with 3 T MR	GLS different significantly between genders: −18.48 ± 3.65% (m) vs. −21.91 ± 3.01% (f) GCS did not differ considerably Aging did not influence GLS or GCS
Andre et al.	2015	150 healthy individuals	FT-CMR with 1.5 T MR	All the following varied significantly: GLS endocardial: −22.2 ± 3.4% (m) vs. −24.6 ± 2.9% (f) GLS myocardial: −20.4 ± 3.1% (m) vs. −22.9 ± 2.7% (f) GRS: 37.9 ± 8.2% (m) vs. 34.8 ± 8.9% (f) GCS endocardial: −26.5 ± 4.2% (m) vs. −27.9 ± 3.7% (f) GCS myocardial: −22.2 ± 3.4% (m) vs. −24.6 ± 2.9% (f)
Aurich et al.	2016	47 healthy individuals	FT-CMR vs. FT-Echo vs. STE	STE: GLS: −15.7 ± 5.0% GCS: −14.6 ± 4.5% GRS: 21.6 ± 13.3% FT-Echo GLS: −13.1 ± 4.0, GCS: −13.6 ± 4.0, GRS: 20.3 ± 9.5, FT-CMR GLS: −15.0 ± 4.0, GCS: −16.9 ± 5.4 GRS: 35.0 ± 10.8 Best agreement was between FT-Echo and FT-CMR for GLS
Bucius et al.	2019	11 healthy individuals + 7 with heart failure	FT-CMRvs. TT-CMRvs. fast-SENC	FT-CMR GLS: −23.5% (−22.0–−25.9) GCS: −26.1% (−21.8–−27.8) TT-CMR GLS: −14.9% (−11.8–−16.9) GCS: −17.8% (−16.4–−19.5) Fast-SENC GLS: −19.4% (17.1–20.7) GCS: −20.3% (16.5–22.3)

Abbreviations: f, female subjects; Fast-SENC, fast Strain-encoding cardiac magnetic resonance; FT-CMR, feature-tracking cardiac magnetic resonance; GCS, global circumferential strain; GLS, global longitudinal strain; GRS, global radial strain; m, male subjects; *n*, number of subjects; TT-CMR, tissue-tagging cardiac magnetic resonance.

**Table 3:** LV myocardial strain assessed by various CMR techniques.

Authors	Ref	Year	*n*	Method	Diagnose	Strain	Findings
El-Saadi et al.	[13]	2022	30	Fast-SENC vs. FT-CMR	AMI	GLS, GCS	Fast-SENC was superior to FT-CMR
Reindl et al.	[60]	2021	232	FT-CMR	AMI	GLS, GCS, GRS	GLS > −14%—independent predictor LV remodeling
Cha et al.	[61]	2019	82	FT-CMR	AMI	GLS	Independent predictor for LV remodeling
Holmes et al.	[62]	2017	141	FT-CMR	AMI	GCS	Independent predictor for LV remodeling
Singh et al.	[73]	2015	18	FT-CMR	AS	GLS, GCS	Higher values
Pozo Osinalde et al.	[64]	2021	N/A	FT-CMR	DCM	GCS	Predictor for LV systolic function recovery
Yu et al.	[63]	2017	48	TT-CMR	DCM	GLS, GCS	Impaired parameters
Moody et al.	[74]	2015	45	FT-CMR	DCM	GLS, GCS	Good agreement
Buss et al.	[65]	2015	210	FT-CMR	DCM	GLS	Independent predictor for outcome
Hor et al.	[78]	2010	233	FT-CMR	DMD	GLS	−13.3%
Pu et al.	[68]	2021	93	FT-CMR	HCM	GCS	Independent predictor for VT
Harrild et al.	[77]	2012	24	FT-CMR	HCM	GLS	Good agreement
Giusca et al.	[70]	2021	214	Fast-SENC	HCM, Athletes’ hearts, AHT	GLS	Disease discrimination
Weise Valdes et al.	[57]	2021	181	fast-SENC	Healthy	GLS, GCS	−20.3%, −19.2%
Erley et al.	[79]	2019	50	fast-SENC	Healthy	GLS, GCS	Good agreement
Taylor et al.	[53]	2015	100	FT-CMR	Healthy	GLS, GCS, GRS	−21.3%, −26.1%, 39.8%
Korosoglu et al.	[66]	2021	1169	Fast-SENC	Heart failure	GLS, GCS	Independent prognostic predictors for outcome
Wu et al.	[75]	2014	30	FT-CMR	LBBB, HCM	GCS	Good agreement
Augustine et al.	[76]	2013	145	FT-CMR	normal	GLS, GCS, GRS	Good agreement

Abbreviations: AHT, arterial hypertension; AMI, acute myocardial infarction; AS, aortic stenosis; DCM, dilated cardiomyopathy; DMD, Duchenne muscular dystrophy; Fast-SENC, fast Strain-encoding cardiac magnetic resonance imaging; FT-CMR, feature-tracking cardiac magnetic resonance imaging; GCS, global circumferential strain; GLS, global longitudinal strain; GRS, global radial strain; HCM, hypertrophic cardiomyopathy; LBBB, left bundle branch block; LV, left ventricle;. N, number of patients; TT-CMR, tissue-tagging cardiac magnetic resonance imaging; VT, ventricular tachyarrhythmias.
